# Packed cell volume Platelet count, PT, PTTK and Fibrinogen concentration of HIV positive patients on antiretroviral drugs

**DOI:** 10.12669/pjms.316.7943

**Published:** 2015

**Authors:** Evarista Odaburhine Osime, Omobolaji Oluwole Oresanja, Benson Uchechukwu Okwara

**Affiliations:** 1 Dr. Evarista Odaburhine Osime, PhD. Department of Medical Laboratory Science, University of Benin, Edo state, Nigeria; 2 Mr. Omobolaji Oluwole Oresanja, BMLS. Department of Medical Laboratory Science, University of Benin, Edo state, Nigeria; 3 Dr. Benson Uchechukwu Okwara, FWACP. Department of Medicine, University of Benin Teaching Hospital, Benin City, Nigeria

**Keywords:** HIV, AIDS, HAART, Haemostatic

## Abstract

**Objective:**

This is aimed at investigating some coagulation and haematologic profile of HIV positive patients on highly active antiretroviral therapy in patients attending clinic at the University of Benin Teaching Hospital.

**Methods:**

This is a correlation study comprising fifty (50) HIV positive patients on HAART between 6 – 12 months as test subjects and fifty (50) HIV positive patients who have not began HAART as control subjects. Five millilitres of blood was withdrawn from each group by venepuncture into ethylene diaminetetracetic and sodium citrate anticoagulant containers. Platelet counts were estimated manually using ammonium oxalate solution, packed cell volume by the microhaematocrit method while Prothrombin Time (PT), Activated partial thrombroplastin time and fibrinogen concentration were done by methods described by Monica Chessbrough.

**Results:**

This is presented as mean ± standard error of mean. There were reduction in PCV and platelet count between test and control subjects although not statistically significant (P> 0.05) while there was a significant increase in PT and PTTK between test and control groups (P<0.05). No significant change was observed in fibrinogen concentration in HIV patients on HAART and those not on HAART.

**Conclusion:**

HAART increases PT and PTTK in HIV infection.

## INTRODUCTION

The human immunodeficiency virus (HIV) and acquired immunodeficiency syndrome (AIDS) has become composite and the rate of bleeding abnormalities is on the increase.^1^ Cytopenias of all major blood cell lines are increasingly recognized among patients infected with the human immunodeficiency virus.^2^ Previous study shows a 3.2 percent prevalence of HIV infection among individuals presenting with unexplained thrombocytopenia or leucopenia lasting for more than four weeks.^2^ HIV-related thrombocytopenia is the most common haemostatic disorder with a high morbidity and is seen to affect people from every risk group independently of age, sex or stage of infection. The advent of antiretroviral drugs has changed the mortality of HIV in the developed world. The first agent to be licensed was zidovudine (AZT), a reverse transcriptase inhibitor.^3^ Haematological changes observed in HIV infection may be as a result of increased peripheral destruction of cells or decreased production of cells because of direct bone marrow suppression by HIV, opportunistic infection, tumour infiltration and treatment therapies.^4^

Thrombocytopenia in AIDS has a complex multifactorial aetiology. There is often immune mediated destruction of platelets usually occurring earlier in the disease process by circulating immunoglobulins, platelet bound IgG, complement or antibody complexes. There is cross-reaction between HIV gp120 and thrombocyte gp3a and between HIV p24 antibodies and platelets. These antibodies are due to a defect in regulation of antibody production characterized by HIV infection; B-cell regulation, activation and response are abnormal as a result of the HIV infection.^5^ Antiphospholipid antibodies and lupus-like anticoagulant of IgG type have been found in HIV positive patients.^6^ This antibody is associated with active opportunistic infection in patients and these patients are seen to have a prolonged activated partial thromboplastin time. The aim of this work therefore was to assess some coagulation and haematologic indices of HIV patients on highly active antiretroviral therapy.

## METHODS

This study was carried out at the PEPAR (Presidential Emergency Plan for AIDS Relief) Laboratories of the University of Benin Teaching Hospital. Subjects comprised one hundred (100) patients randomly selected. Test subjects comprised of fifty (50) HIV confirmed HIV sero-positive patients on highly active antiretroviral drugs (HAART) and fifty (50) infected HIV seropositive patients who have not commenced HAART as controls.

**Table-I:** Comparison of Haemostatic parameters found in the HIV patients.

Parameters	Control subject (mean±SEM) n=50	Test subject (mean±SEM)n=50	P value	Level of Significance
PCV (%)	27.75 ± 0.21	27.29 ± 0.89	0.149	P > 0.05
Platelet count(x10^9^/L)	92 ± 1.73	86 ± 1.81	0.371	P > 0.05
PT (s)	22.62 ± 1.16	30.30 ± 2.56	1.191	P < 0.05
PTTK (s)	39.53 ± 2.02	50.07 ± 2.20	0.000013	P < 0.001
Fibrinogen (g/l)	3.70 ± 0.19	4.34 ± 0.24	0.0510	P > 0.05

### Ethical Clearance

This was obtained from the Ethics and Research Committee of the University of Benin Teaching Hospital, Benin City.

### Inclusion and Exclusion Criteria

Inclusion criteria included HIV positive patients who have been confirmed to having the disease on antiretroviral drugs and HIV positive patients that have not commenced antiretroviral therapy while exclusion criteria were patients with haematological and other abnormalities like haemophilia, liver disease, renal diseases, and inflammatory conditions that could interfere with haemostatic mechanism.

### Sample Collection and Methods

Five millilitres of whole blood was collected using a monovette vacutainer syringe. The blood was deposited into an ethylenediaminetetraacetic acid (EDTA) anticoagulated tube used for the analysis of packed cell volume and platelet count and into a sodium citrate anticoagulated tube. The citrate anticoagulated sample was centrifuged highly for 5 minutes at 3000 rpm. The citrated plasma was separated aseptically and used for PT, PTTK and fibrinogen concentration. Determination of packed cell volume (PCV) was carried out using the microhaematocrit method as described by Dacie and Lewis,^7^ platelet counts were estimated manually using the improved Neubauer Counting Chamber. Fibrinogen concentration, PT and PTTK were assayed using Diagen Diagnostics Reagent (Diagen Reagents Ltd, Thame, UK). Test procedures were conducted according to the instructions in the manufacturer’s standard operating manual.

### Statistical Analysis

The effect of antiretroviral drugs in 50 HIV infected subjects on HAART (test) were compared to 50 HIV infected subject not on HAART (control). The result obtained were presented as mean ± standard error of mean. All calculations were done using the SPSS – V16 Statistical Software Package for Analysis of data. Comparisons were done using the student’s paired t-test and differences were considered to be statistically significant at an error probability of less than or equal to 0.05 (P≤0.05).

## RESULTS

This shows a reduction in PCV and platelet counts between test and control subjects, although not statistically significant (P > 0.05). The PT and PTTK values were observed to increase significantly (P < 0.05) between HIV patients on HAART (test) and those not on HAART (controls) while there was no significant change in fibrinogen concentration between test and control subjects.

## DISCUSSION

Haemorrhage is a major risk factor in a lot of immunosuppressive disorders such as the dreaded HIV and AIDS. This haemorrhage may occur as a result of HIV-related opportunistic infections or as a consequence of antiretroviral therapies used in the treatment of the disease.

In this study, the packed cell volume of all subjects were observed to be reduced. Control subjects had a slightly higher levels of PCV than the test subjects although not statistically significant. It has been observed that anaemia is a common finding in patients with HIV infection, particularly in individuals with more advanced HIV disease.^9^ Investigation from a longitudinal study of HIV disease found a very high incidence rate of anaemia in HIV patients.^10^ HIV infection alone, without other complicating illness, may produce anaemia in some patients. Immunoreactive hormone such as erythropoietin levels in HIV infected patients showed that levels of the hormone failed to rise commensurately with increasing anaemia, suggesting that insufficient amounts of erythropoietin may be one cause of this anaemia.^11^ Studies have also suggested that soluble factors in the serum of HIV-infected patients may inhibit haematopoiesis also that direct HIV infection of marrow progenitor cells may play a role in producing anaemia and other haematologic abnormally associated with HIV infection.^12^,^13^

Thrombocytopenia is a common occurrence in HIV infection. In our study, we observed a mean platelet count of 92±1.73 in our controls and 86.0±1.81 in our test. This could be due to immune mediated destruction of platelets, impaired haematopoiesis and the toxic effects of HAART. Another school of thought in thrombocytopenia occurring a lot in HIV patients is that circulating immune complexes are non-specifically deposited on platelet membranes resulting in reticuloendothelial clearance.^14^,^15^ Regardless of the actual mechanism responsible for platelet destruction, thrombocytopenia in HIV-infected patients may be compounded by impaired ability to produce platelets in sufficient numbers.^16^

Also, there exist some abnormalities especially in the fluid phase of the coagulation cascade which may produce bleeding in the HIV patient. This can invariably leads to a prolonged partially activated thromboplastin time test, the production of a hepic anticoagulant, anticardiolipin antibodies and several abnormalities.^17^ This could be responsible for the increase in PT and PTTK observed in this study in the control subjects and much more higher values observed in the test subjects. This may be attributed to the drugs used in the management of these patients because a number of medications commonly used in treatment of HIV infection can cause coagulation abnormalities.^18^

The fibrinolytic system acts as a balance to the coagulation system, preventing excess clotting by breaking down fibrin. In this study, we observed a fibrinogen level of 4.34±0.24 g/l in the test subjects and 3.70±0.19 g/l in the control subjects. These values are not significantly different when compared.

## CONCLUSION

HAART increases PT and PTTK in HIV infection. It is worthy of note that despite the potential side effects of these drugs, they may be essential for treatment of HIV infection or its complications, so they should not necessarily be avoided. Instead, people should be aware that side effects abound and efforts should be made to monitor and treat them.
